# Bioinformatics designing of an mRNA vaccine for Mokola virus (MOKV) using immunoinformatics as a secure strategy for successful vaccine development

**DOI:** 10.1186/s12865-024-00668-2

**Published:** 2024-11-20

**Authors:** Elijah Kolawole Oladipo, James Akinwumi Ogunniran, Oluwaseyi Samuel Akinpelu, Tosin Omoboyede Omole, Stephen Feranmi Adeyemo, Boluwatife Ayobami Irewolede, Bamidele Abiodun Iwalokun, Olumide Faith Ajani, Helen Onyeaka

**Affiliations:** 1 Division of Vaccine Design and Development, Helix Biogen Institute, Ogbomoso, Oyo State Nigeria; 2 Division of Genome Sciences, Helix Biogen Institute, Ogbomoso, Oyo State Nigeria; 3 Department of Microbiology, Laboratory of Molecular Biology, Immunology and Bioinformatics, Adeleke University, Ede, Osun State 232104 Nigeria; 4 Department of Chemical Engineering, University of Birmingham, Edgbaston, Birmingham, B12 2TT UK; 5 Molecular Biology & Biotechnology Department, Nigerian Institute of Medical Research, Lagos, 101212 Nigeria; 6 African Centre for Disease Control and Prevention (African CDC), Addis Ababa, Ethiopia

**Keywords:** Immunoinformatics, Vaccination, Mokola, CD8 + T lymphocytes, CD4 + T lymphocytes

## Abstract

The Mokola Virus belongs to the family Rhabdoviridae and is genotype 3 of the Lyssavirus genera. A small number of cases of animal and human encephalomyelitis, mainly scattered over sub-Saharan Africa, have been linked to the Mokola Virus (MOKV). Currently there is no vaccine to protect against MOKV infection in people or animals. It has been proven that rabies vaccination does not confer immunity against MOKV infection, even though MOKV and the rabies virus are related. Using immunoinformatics approaches, this study designed an mRNA vaccine that can protect against all the five glycoproteins of the Mokola virus. NCBI was used to obtain the viral sequences, which were then screened for antigenicity, allergenicity, toxicity, B-cell epitopes, CD8 + T lymphocytes (CTL), and CD4 + T lymphocytes (HTL). These epitopes were used in the construction of the vaccine. Some extra co-translational residues were added to the mRNA vaccine construct. Its molecular weight is 129.19083 kDa, and its estimated pI is 8.58. It interacts rather steadily and with limited deformability with TLR 3, among other human innate immune receptors. Overall, the results show that the produced candidate vaccine is non-allergen, non-toxic, and can elicit T–cell and B–cell immune responses. These findings can further be subjected to in-vivo and in-vitro techniques for validation.

## Introduction

Mokola virus (MOKV) is an RNA virus related to the rabies virus that has been identified from mammals in sub-Saharan Africa on occasion. Most of the isolates have originated from domestic cats displaying symptoms that are typical of an infection with the rabies virus [1]. The Mokola virus (MOKV) is a member of the Rhabdoviridae family, specifically the genus Lyssavirus. There are four lyssaviruses in Africa, including MOKV. According to von Teichman et al.. (1998), the remaining three viruses are the Lagos bat virus, the Duvenhage virus, and the Conventional Rabies Virus. In 1968, MOKV was initially isolated in Nigeria from three Crocidura species shrews that were discovered in the Mokola woodland in Ibadan, Oyo State. The virus was found to share similarities with the rabies virus both morphologically and serologically. Since MOKV was first discovered, small animals and domestic cats in sub-Saharan Africa have been the primary hosts of the virus [2].

The single-stranded, non-segmented MOKV genome is made up of five proteins that are encoded by the genomes including matrix protein (M), phosphoprotein (P), nucleoprotein (N), transmembrane glycoprotein (G), RNA-dependent RNA polymerase (L) [3]. MOKV’s transmembrane glycoprotein (G) mediates its entrance into host cells. G first causes virion endocytosis by binding to cellular receptors. Then, G experiences a conformational shift from its pre- to post-fusion state in the acidic endosomal environment, which catalyzes the union of the viral and endosomal membranes [4]. According to Mercier et al.. (1997), the matrix protein (M or M2) is involved in the morphology of virion. The nucleoprotein (N) tightly encapsulates the genome, serving as a template for the polymerase complex that consists of the cofactor phosphoprotein (P or M1) and the RNA-dependent RNA polymerase (L). The other three proteins in the ribonucleocapsid structure are involved in transcription and replication [3].

Currently no vaccination for humans or animals’ guards against MOKV infection [1]. Although MOKV and the rabies virus are linked, it has long been known that receiving the rabies vaccine does not provide immunity against MOKV infection [2]. Vaccinations are the most effective boon for humanity in preventing the spread of infectious diseases [5]. The impact of vaccination on the economic viability of the healthcare system is huge since it lowers the treatment costs of infectious diseases [5]. Additionally, vaccines also aid in reducing the impact and risk of outbreaks [6]. We are currently in the era of mRNA vaccinations because the groundwork research was laid over three decades ago [7].

mRNA vaccines have been demonstrated as a powerful alternative to traditional conventional vaccines because of their high potency, safety and efficacy, capacity for rapid clinical development, and potential for fast, low-cost manufacturing [5]. Successful vaccine design against several viruses have been studied and is critical for controlling infectious diseases and enhancing public health. Effective vaccines must elicit robust immune responses, providing both immediate and long-lasting protection. The key strategies used in vaccine design and development include the identification of immunogenic epitopes, optimization of antigen presentation, and the use of new delivery systems, such as nanoparticles, to enhance immunogenicity and stability [6, 8]. Furthermore, employing reverse vaccinology and systems biology approaches allows for the identification of potential vaccine candidates based on viral genomes [9]. The development of mRNA vaccines, exemplified by the rapid deployment of COVID-19 vaccines, has revolutionized vaccine design and development, demonstrating the ability to induce strong immune responses with a favorable safety profile [10, 11]. Continued advancements in adjuvant technology and understanding of viral pathogenesis will further improve vaccine efficacy and accessibility.

mRNA vaccines also have a broad range of potential for correcting abnormalities such as protein expression and higher therapeutic efficacy because mRNA vaccines translate more gradually into encoded proteins and peptides to activate long-lasting effects than peptide-based vaccines [12]. According to this viewpoint, we used integrated approaches to vaccine design by joining the B-cell, CTL, and HTL epitopes found on the virus’s five structural glycoproteins based on several parameters. Then, we used in-silico immune simulations to validate the final proposed vaccine construct by injecting the vaccine and tracking the immune response.

## Methodology

### Retrieval of the target protein sequence

The protein sequences of all five (5) proteins of Mokola Virus available on the National Center for Biotechnology Information (NCBI) [13] as of November 24, 2023, were simultaneously retrieved for appropriate analysis. NCBI is a large and heterogenous database that houses biological dataset and can be retrieved in the FASTA format.

### Prediction of cytotoxic T-cell lymphocytes (CTLs) epitopes

MHC class-I is an important component of the immune system for generating a CD8^+^ T-cell response, and it is crucial in designing T-cell-engaging vaccines against viruses [14]. NetCTL v1.2 [15] and NetMHCpan 4.0 [16] servers were used for the prediction of the CTL epitope. These servers use diverse algorithms to scan out epitopes that can stimulate CD8^+^ T-cell response. The consensus epitope across the two servers was selected for the vaccine construct.

### Prediction of helper T-cell lymphocytes (HTLs) epitopes

MHC class II is an important component of immune system for generating humoral and cellular immune responses [17]. MHC-II binding prediction on IEDB [18] and NetMHCIIpan 4.0 server [19] were used to predict the epitopes that bind to the MHC class-II and the consensus HTL epitopes across the two servers were selected for the vaccine construct.

### Prediction of linear B- lymphocytes (LBL) epitopes

B-cells are a component of the immune system capable of producing long-term defense against infections and antigens. B-cell prediction is very important in the development of epitope vaccines [20]. Three immunoinformatics tools, ABCPred [21], B-cell epitope prediction on IEDB [18], and Bepipred [22], were used to predict the B-cell epitopes in the MOKV glycoprotein sequences. The consensus epitopes across the three servers were selected for the vaccine construct.

### In-silico mRNA vaccine construction

The selected epitopes were joined with some additional co-translational residues to form the mRNA vaccine construct as suggested by Oluwagbemi et al.. (2022) [11]. 5′ cap was placed first to ensure the stability and functionality of the mRNA vaccine construct. The 5′ UTR helps in mRNA stability and translation. The Kozak sequence forms the start codon. An adjuvant called Human Beta Defensin (HBD1) was used to co-stimulate the vaccine’s capacity to produce an antigenic response [23]. GPGPG linkers was used to joined both adjuvant and the HTL epitopes; HTL epitopes were joined with the LBL epitopes by (EAAK) linkers; and LBL and CTL epitopes were joined by AAY. A tissue plasminogen activator (tPA), an MHC I-targeting domain (MITD), and a signal peptide were joined with the vaccine construct. tPA was used to eject the translated epitopes into the circulatory system [24]. MITD helps to attach the CTL epitopes to the endoplasmic reticulum’s MHC-I compartment. 3′ UTR and Poly-A tail were added at the end of the vaccine construct to improve mRNA stability and translation.

### Prediction of antigenicity, allergenicity, and toxicity of vaccine construct

The antigenicity, allergenicity, and toxicity of the mRNA vaccine construct was predicted using different bioinformatics tools. Antigenicity was predicted using VaxiJen 2.0 [25]. The allergic reaction of the vaccine construct was predicted using AllerTop 2.0 [26] while the toxicity of the vaccine construct was predicted by ToxinPred 2 [27] to be sure of the safety of the vaccine construct in terms of the antigenicity, allergenicity, and toxicity.

### Prediction of physicochemical properties of the vaccine candidate

The physicochemical properties of the vaccine were examined using the ExPASy ProtParam tool [28]. This tool is a public online server that is used in determining the physical and chemical properties of the final vaccine construct. Properties such as the construct’s length, molecular weight, instability index, theoretical isoelectric point (pI), aliphatic index, estimated half-life, and the grand average of hydropathicity (GRAVY) were predicted. The solubility of the vaccine construct was also checked to examine the hydrophobicity and hydrophilicity of the amino acid residues using the Protein–Sol server [29].

### Prediction of the secondary structure

The secondary structure of the vaccine construct is essential as it helps to give an information about the amino acid interaction in the construct [30]. The SOPMA server was used to predict the secondary structure of the vaccine construct [31]. This tool optimizes multiple predictions from various alignments, employing the technique of BLAST (Basic Local Alignment Search Tool) to enhance and characterize the prediction of the vaccine’s secondary structure.

### Tertiary structure prediction, refinement, and validation

The Swiss model [32] was used to predict the 3-D structure of the vaccine construct while it was refined using the GalaxyRefine server. The amino acids in the favorable region were revealed by the Ramachandran plot, predicted using ProCheck [33].

### Conformational B-cell epitopes prediction

Linear and conformational B-cell epitope which serve as the antigenic determinants of B-cells was predicted by ElliPro, a tool that deduced antibody epitopes using structure-based [34].

### Molecular docking of the vaccine construct

According to Takeuchi and Akira (2010) [35], Toll-Like Receptors (TLRs) are proteins that stimulate an immune response that results in the production of type I interferons (IFNs) and pro-inflammatory cytokines. It has been proved that TLR-3 receptors may identify viral or microbial nucleic acids [36]. Therefore, the vaccine construct’s tertiary structure was docked to TLR-3 (retrieved from the RCSB PDB database with ID “1ziw”) using the Cluspro server [37]. Molecular docking predicts the binding energy between the ligand and receptor. The binding energy is based on their scoring function.

### Molecular dynamics simulation analysis

Molecular dynamics simulation is a computational tool that shows the detailed atomic behavior of proteins at a good time [38]. iMOD was used to reveal the interaction between the vaccine construct and the TLR-3 complex. The tool operates by showing the normal mode analysis (NMA) in internal (dihedral) coordinates that naturally reproduce the joint functional motions of biological macromolecules and generate feasible transition pathways between two homologous structures, also with large macromolecules [39].

### Immune response simulation

Immune response simulation offers the chance to test the overall immunogenicity of a generic protein sequence. C-immsim tool was used to predict the immune response simulation of the vaccine [40]. This server simulated the body’s response based on its ability to elicit immunological responses against the injected vaccine. Additionally, it estimated the number of immune cells, such as helper T-cells, measured the amount of interferon, cytokines, and antibodies produced against injected vaccines, and observed other immunological reactions. The server’s immune response simulation parameters were set to default for the prediction.

**Fig. 1:**
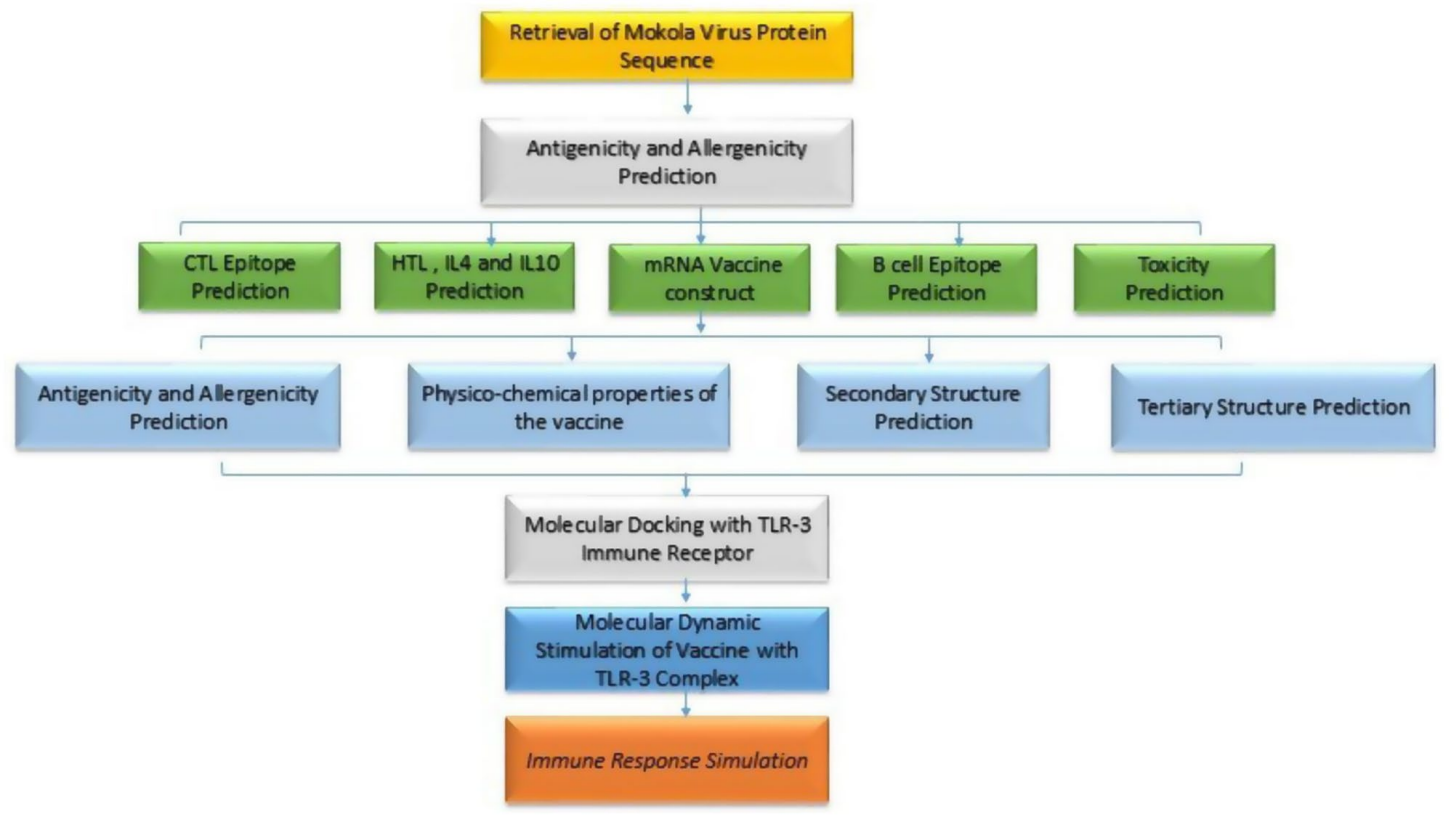
Study outline for the Mokola vaccine design

A structured outline was used for this study, which is illustrated in Fig. 1. This structure shows all the steps involved in designing the mRNA vaccine.

## Result

### Retrieval of protein sequence

The accession numbers of the five (5) retrieved sequences is shown in Table 1. They include YP_142350.1, YP_142351.1, YP_142352.1, YP_142353.1 and YP_142354.1, all from Africa, Nigeria to be precise.

**Table 1:** Prediction of the antigenicity of the protein sequence

S\*N*	Accession number	Protein	VaxiJen	Antigenic pro
1	YP_142350.1	Nucleoprotein	0.4529	0.676953
2	YP_142351.1	Phospho-protein	0.4015	0.776712
3	YP_142352.1	Matrix protein	0.4581	0.867012
4	YP_142353.1	Transmembrane Glycoprotein	0.4010	0.702098
5	YP_142354.1	RNA polymerase	0.4298	-

### Prediction of MHC class I binding epitopes

All five Mokola virus glycoproteins were subjected to CTL prediction. Five CTL epitopes passed the analysis carried out on them, which includes the antigenicity, toxicity, and allergenicity as shown in Table 2 below.

**Table 2:** Result of the CTL prediction

Protein	Epitopes	Antigenicity	Allergenicity	Toxicity
Nucleoprotein	WSTIPNFRF	Antigenic	Non-allergen	Non-toxic
Phosphoprotein	LIQEDINSY	Antigenic	Non-allergen	Non-toxic
Matrix protein	SFKILRHIL	Antigenic	Non-allergen	Non-toxic
Transmembrane Glycoprotein	PSCETNHDY	Antigenic	Non-allergen	Non-toxic
RNA polymerase	LSSCGNAGY	Antigenic	Non-allergen	Non-toxic

### Prediction of MHC class II binding epitopes

All five Mokola virus proteins were subjected to HTL prediction. Five HTL epitopes passed the analysis carried out on them, which includes the antigenicity, toxicity, allergenicity, IL-4, and IL-10 as shown in Table 3 below.

**Table 3:** Result of the HTL prediction

Protein	Epitopes	Antigenicity	Allergenicity	Toxicity	IL 4	IL 10
Nucleoprotein	SDKIVFKVNNQVVSL	Antigenic	Non-allergen	Non-toxic	Inducer	Inducer
Phosphoprotein	DMSRLRIEDKSRRTK	Antigenic	Non-allergen	Non-toxic	Inducer	Inducer
Matrix protein	HGPLEGEELEYSQEI	Antigenic	Non-allergen	Non-toxic	Inducer	Inducer
Transmembrane Glycoprotein	RKHFRPTVAACRDAY	Antigenic	Non-allergen	Non-toxic	Inducer	Non-Inducer
RNA polymerase	TSIDIKNDRVYFKDP	Antigenic	Non-allergen	Non-toxic	Inducer	Inducer

### Prediction of linear B-cell lymphocyte (LBL) epitopes

All five Mokola virus glycoproteins were subjected to B-cell prediction. Five B-cell epitopes from all the protein passed the analysis carried out on them, which includes the antigenicity, toxicity, and allergenicity as shown in Table 4 below.

**Table 4:** Result of the LBL cell prediction

Protein	Epitopes	Antigenicity	Allergenicity	Toxicity
Nucleoprotein	HHTLMTTHKMCANWST	Antigenic	Non-allergen	Non-toxic
Phosphoprotein	TKSIQIQTEPTASVSS	Antigenic	Non-allergen	Non-toxic
Matrix protein	DDIWMPPPEYVPLTQV	Antigenic	Non-allergen	Non-toxic
Transmembrane Glycoprotein	KIEKWTPIDM	Antigenic	Non-allergen	Non-toxic
RNA polymerase	EVTLDAPQLFDFPDIS	Antigenic	Non-allergen	Non-toxic

### mRNA universal vaccine construction for Mokola virus

**Fig. 2:**
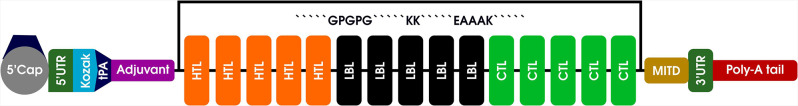
mRNA primary vaccine construct

A total of fifteen epitopes were selected for the vaccine candidate after carrying out different analyses on them. These epitopes include 5 B-cell epitopes, 5 CTL epitopes, and 5 HTL epitopes. Residues such as the 5′ cap, 5′ UTR, Kozak sequence, and tPA (signal peptide) were included in the construction of the primary construct, which were added with the adjuvant (β defensin 1) and then joined to the HTL epitope with the aid of the GPGPG linkers to form the universal mRNA vaccines as shown in Fig. 2 below. HTLs were linked with LBL Epitopes with the linkers KK and EAAAK (LBL to CTL and intra-CTL), respectively, based on their compatibility. The other terminal end of the constructed CTL epitopes was linked together by EAAAK linkers.

### Prediction of antigenicity, allergenicity, and toxicity

The antigenicity score of the vaccine construct is 0.5152 which is above the default threshold level (0.4) of the VaxiJen sever, which shows that the vaccine is capable of stimulating antibodies. The Allergenicity and Toxicity result also shows that the vaccine is non-allergic and non-toxic. This result reveals that the vaccine is good and safe if administered.

### Physicochemical properties of the vaccine construct

The vaccine design has a molecular weight of 129190.83 kDa and is composed of 1162 amino acids, according to the prediction of its physicochemical parameters. With an estimated pI of 8.58, the mRNA vaccine design was predicted to have a relatively basic character. In vitro, the vaccine is predicted to have a half-life of approximately 30 h in mammalian reticulocytes. The vaccine’s instability index was 39.43 showing that the vaccine is stable. The construct’s aliphatic index was 74.81, indicating that it is thermostable. The mRNA construct’s hydropathicity was demonstrated by its negative GRAVY score of -0.479.

### Prediction of the solubility properties of the vaccine construct

**Fig. 3:**
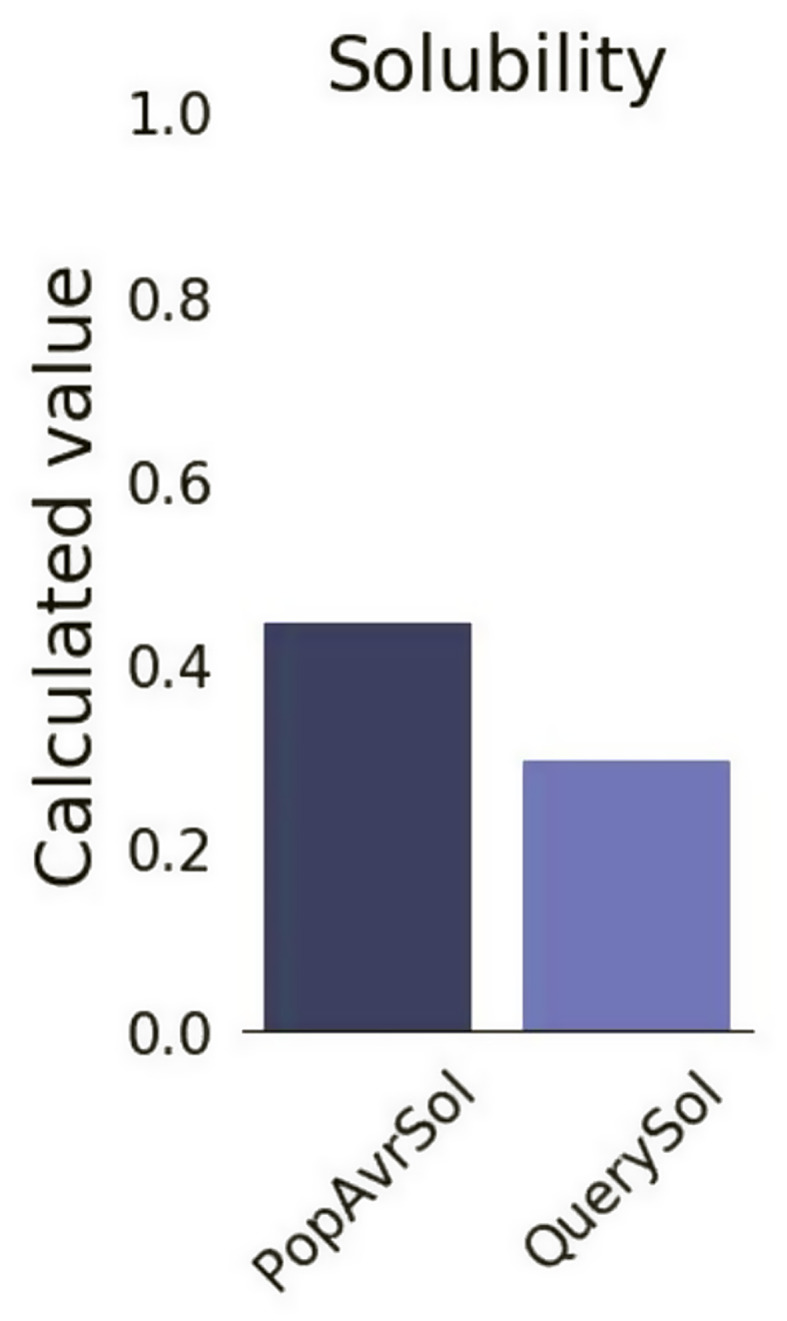
Solubility score of the vaccine construct

A subsequent Protein Sol assessment indicated the vaccine construct to be soluble, with a solubility score of 0.297 (Fig. 3).

### Prediction and evaluation of the mRNA vaccine’s secondary structure

**Fig. 4:**
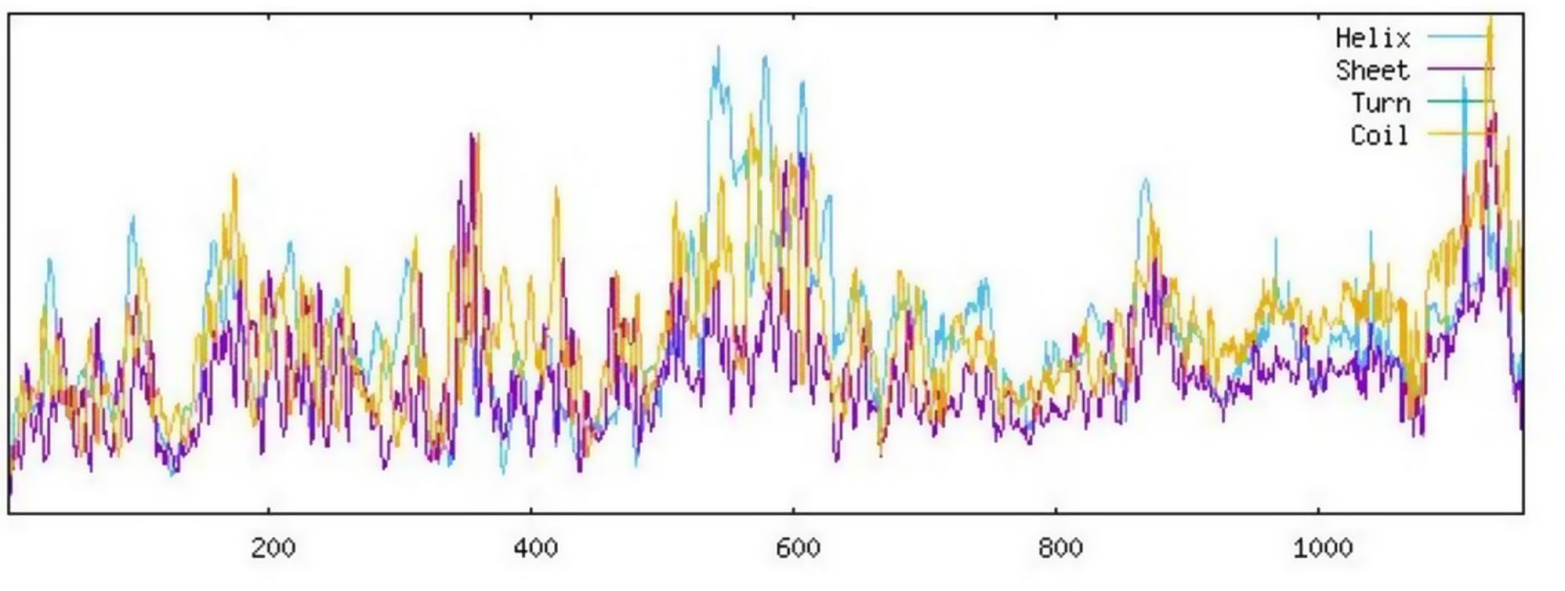
Secondary structure of the vaccine construct

The prediction of the secondary structure of the vaccine construct was carried out using the SOPMA server. The result of the structure was 39.16% alpha-helix, 11.7% extended strands, 0.00% beta-turn, and 49.05% random coils which revealed that the structure was stable as shown in Fig. 4. This result confirmed that the secondary structure of the vaccine construct had high globular conformation, flexibility, and stability. Table 5 explains the properties, number of residues and their percentages respectively.

**Table 5:** Secondary structure of the vaccine construct

Properties	Number of residues	Percentage
Alpha helix (Hh)	455	39.16%
3_10_ helix (Gg)	0	0.00%
PI helix (Ii)	0	0.00%
Beta bridge (Bb)	0	0.00%
Extended strand (Ee)	127	11.7%
Beta turn (Tt)	0	0.00%
Band region (Ss)	0	0.00%
Random coil (Cc)	570	49.05%
Ambiguous states (?)	0	0.00%
Other states	0	0.00%

### Tertiary structure prediction, refinement, and validation

**Fig. 5:**
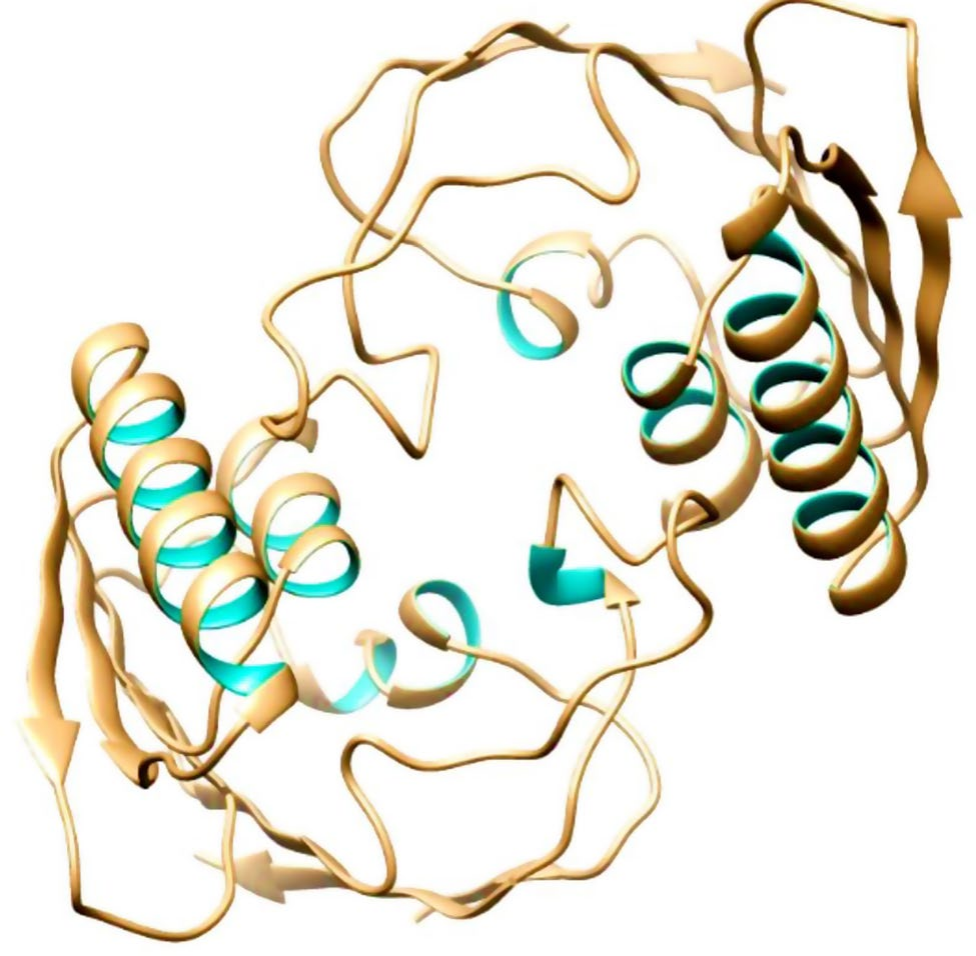
Tertiary structure of the mRNA vaccine

**Fig. 6:**
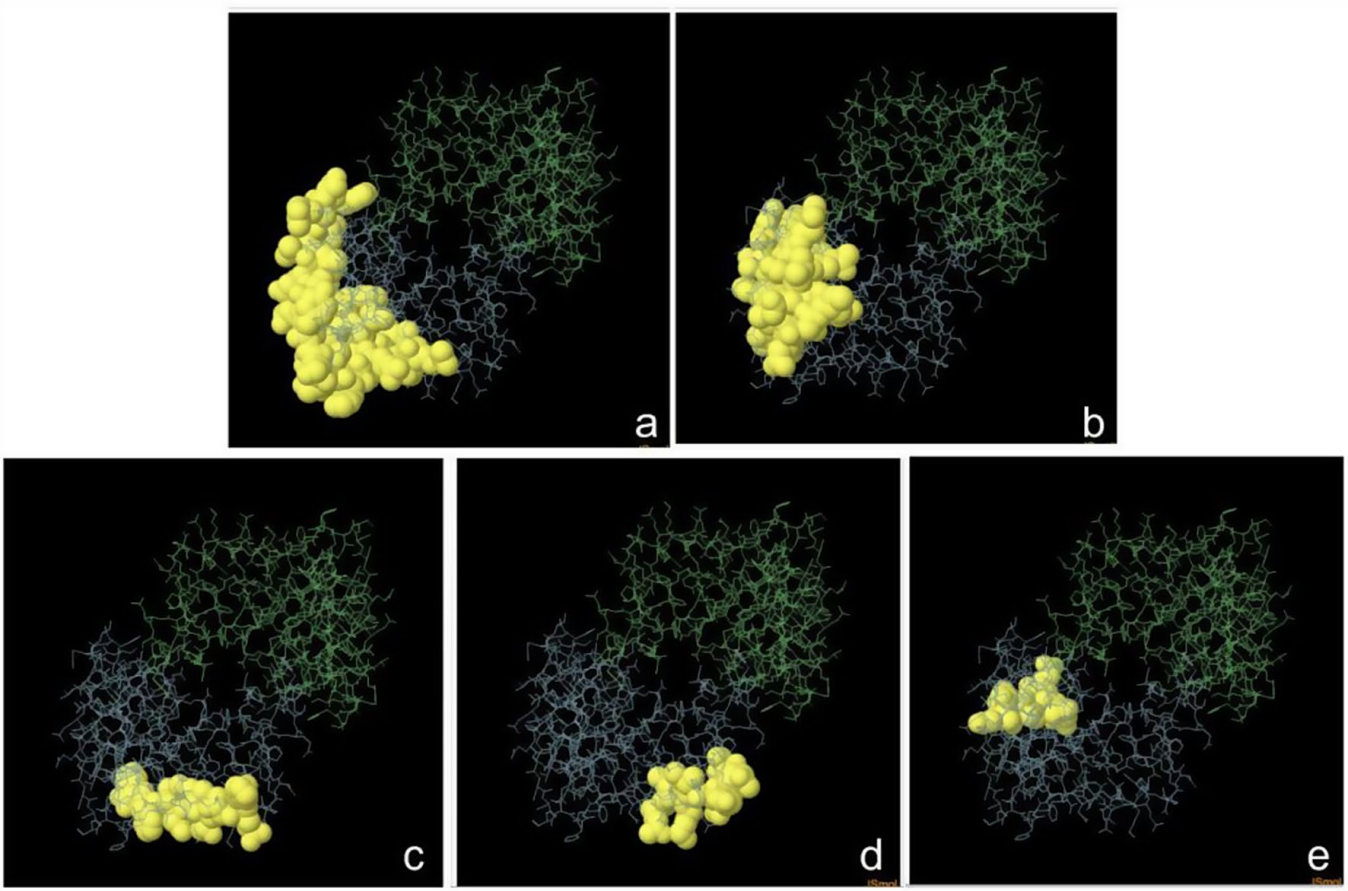
The 3-D model of the 5 predicted conformational B-cell epitopes. The yellow regions are the conformational B-cell epitopes, while the grey regions are the residue remnant. (**a**) 32 residues with a pI score of 0.646 (**b**) 23 residues with a score of 0.684 (**c**) 9 residues with a score of 0.742 (**d**) 8 residues with a score of 0.724; (**e**) 7 residues with a score of 0.657

**Fig. 7:**
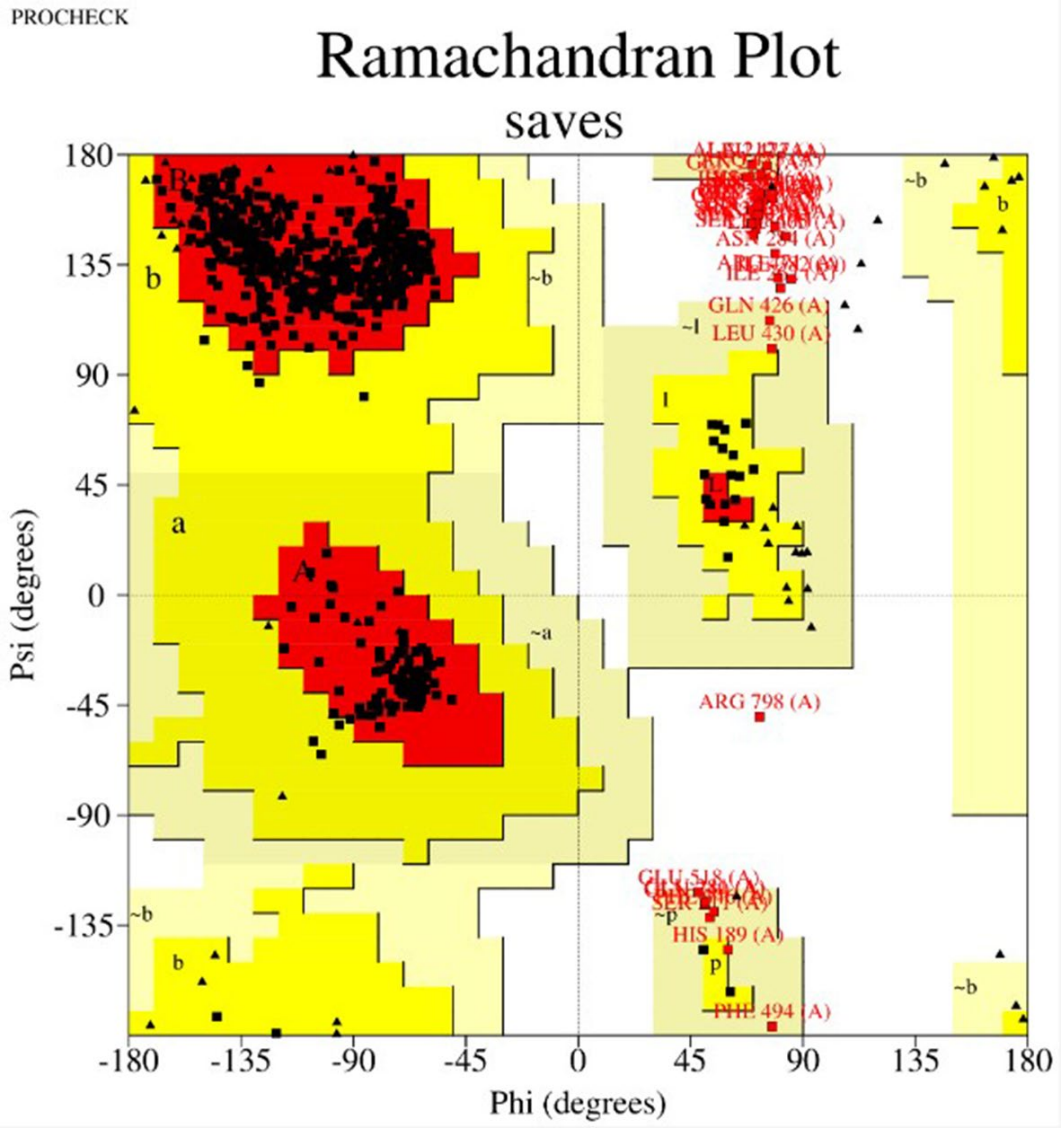
Ramachandran residues score plot

Swiss Model was used to predict the mRNA vaccine candidate’s tertiary structure as shown in Fig. 5. Figure 6 shows The 3-D model of the 5 predicted conformational B-cell epitopes. Five conformational B-Cells epitopes were predicted, for the evaluations of the 3D structure. Ramachandran plot score statistics revealed that 76.0% of the amino acid residues are in the favored region, 22.9% in the additional allowed region, 1.0% in the generously allowed region, and 0.0% in the disallowed as shown in Fig. 7.

### Molecular docking of vaccine with toll-like receptor (TLR)

**Fig. 8:**
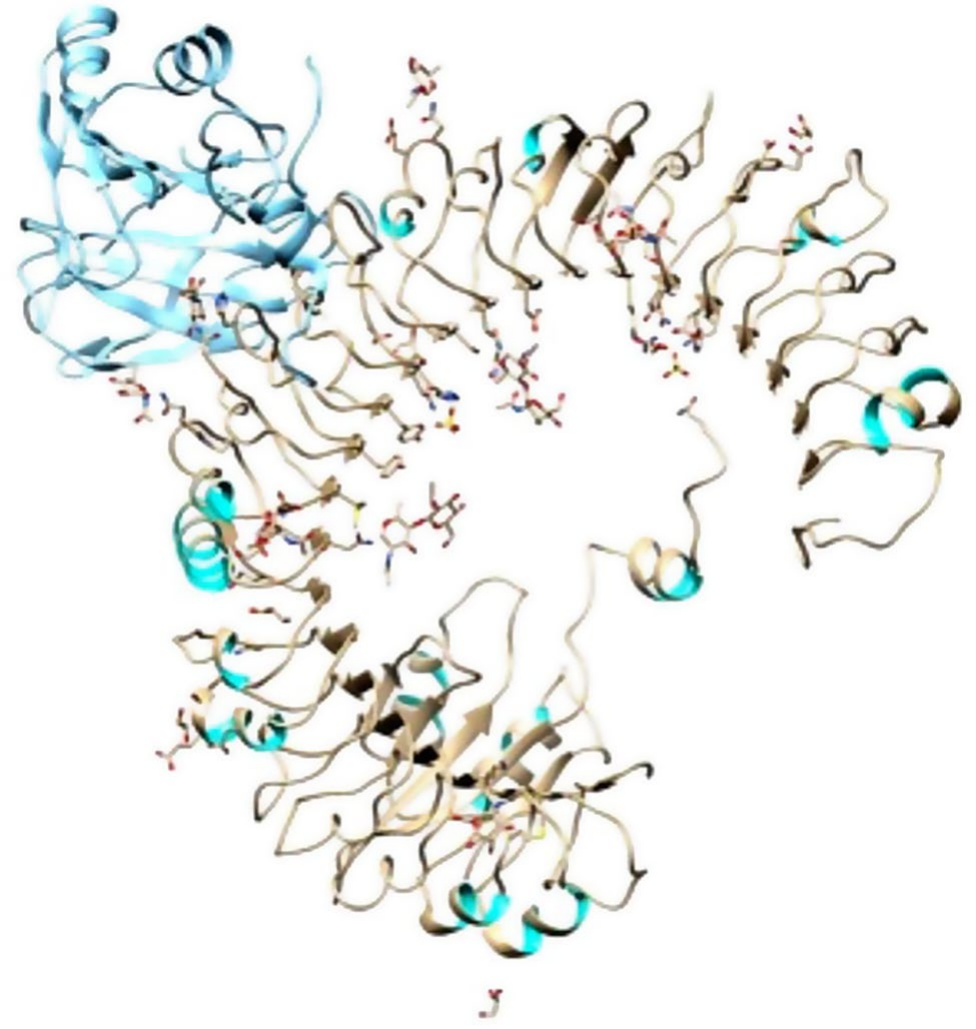
3-D model of vaccine construct and TLR-3 complex

The evaluation of the interaction between the vaccine candidate and potential receptor was examined through molecular docking, utilizing the Cluspro server. Nine models were generated, but the best of it all in respect to low energy and large size was selected as it indicates a good interaction between the receptor and ligand. The results of the molecular docking are presented below (Fig. 8).

### Molecular dynamics simulation

**Fig. 9:**
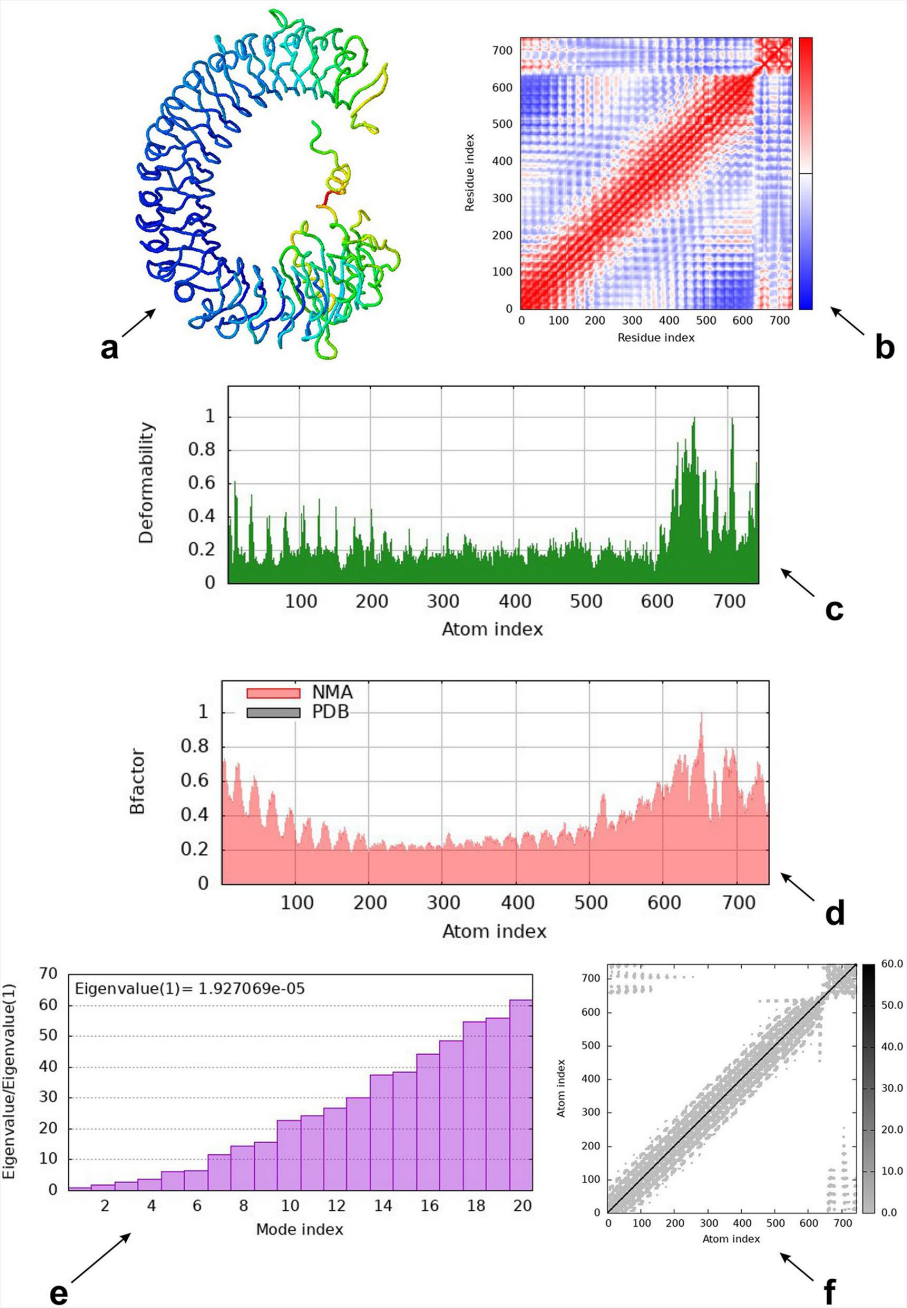
Molecular dynamics simulation (**a**) Spin prediction of the ligand-receptor interaction (**b**) Covariance map of the ligand-receptor interaction (**c**) Deformability B-factor region of the ligand-protein interaction (**d**) Mobility B-factor of the ligand-protein interaction (**e**) Eigenvalues of the ligand-receptor interaction (**f**) Elastic network of the ligand-protein interaction

The molecular dynamic simulation of the mRNA vaccine construct with TLR-3 revealed the result of the spin structure predicted for the Ligand–Receptor Interaction, the deformability graph, the B-factor mobility, the NMA mobility eigenvalues, which are 8.699533 × 10^− 7^, the elastic network, and the covariance matrix analysis, which explained the atomic pairs of the complex (Fig. 9). The red color indicates the correlated portion, while the blue and white colors indicate the non-correlated and uncorrelated portions, respectively.

### Immune response simulation

**Fig. 10:**
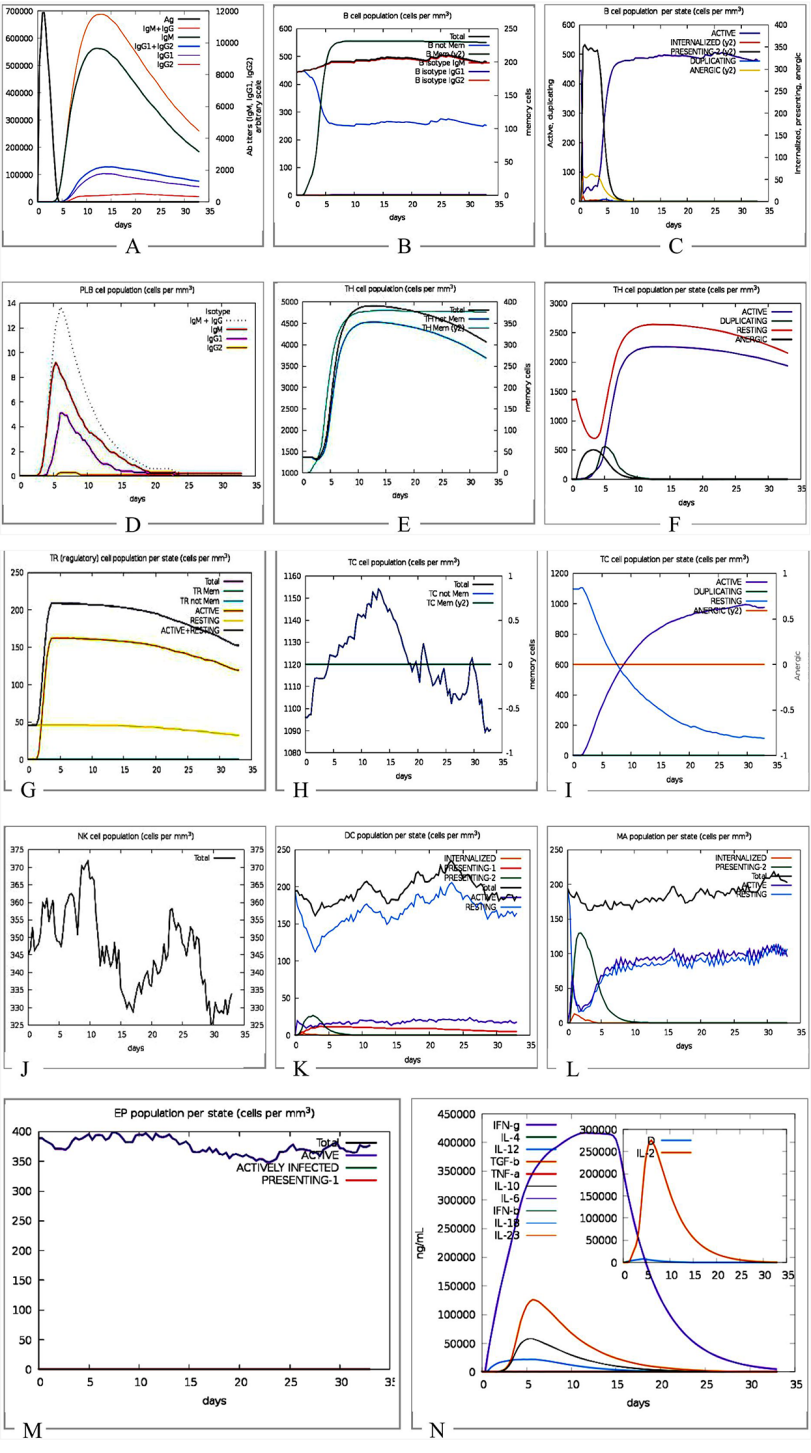
Immune simulation prediction: (**a**) Antigens and immunoglobulins. Antibodies are sub-divided per isotype. (**b**) Lymphocytes B total count memory cells. (**c**) B lymphocytes population per entity-state (i.e., showing counts for active, presenting on class-II, internalized the Ag, duplicating and anergic. (**d**) CD4 T-helper lymphocytes count. Total and memory counts were shown on the plot. (**e**) CD4 T-helper lymphocytes count sub-divided per entity-state (i.e., active, resting, anergic, and duplicating). (**f**) Plasma B lymphocytes count sub-divided per isotype (**g**) CD4 T-regulatory lymphocytes count. (**h**) CD8 T-cytotoxic lymphocytes count. Total and memory shown. (**i**) CD8 T-cytotoxic lymphocytes count per entity-state (**j**) Natural killer cells (total count). (**k**) Dendritic cells. (**l**) Macrophages. Total count, internalized, presenting on MHC class-II, active and resting macrophages. (**M)** Epithelial cells. The total count is broken down to active, virus-infected, and presenting on class-I MHC molecule. (**N)** Cytokines. Concentration of cytokines and interleukins. D in the inset plot is a danger signal

The immune simulation demonstrates the interaction and activations of cells in the innate and adaptive systems. The C-Immsim results in Fig. 10 revealed that the proposed vaccine is capable of stimulating dendritic cells, macrophages, epithelial cells are on a single dose of the multi-epitope vaccine construct. It also shows that the vaccine is capable of producing CD4 T-helper lymphocytes, CD8 + lymphocytes, and NK cells within the first few days of exposure. Also, stimulate B lymphocytes and plasma B lymphocytes.

## Discussion

The Mokola virus (MOKV) is a lyssavirus linked to rabies that seems to be exclusive to Africa. Since the virus’s discovery in 1968, only 24 cases of which two involve humans have been documented. Current commercial vaccinations do not protect MOKV, and the virus’s reservoir host is unclear [41].

All five antigenic proteins of the Mokola virus were used to predict the CTL, HTL, and B-cell epitopes that were used in the vaccine using different immunoformatics techniques. Immune response by successful vaccine development seems to involve elicitation of both humoral and cellular components of immunity [42]. Also, the immune response to antigen is mainly dependent on, how it gets recognized by B-cells and T-cells [43]. We identified epitopes that can elicit B-cells and T-cells in each protein so that both innate and adaptive immunity can be generated with exposure to vaccine construct.

CTL, HTL, and B-cell epitopes and some compounds were added and joined with appropriate linkers according to Oluwagbemi et al.. (2022) [11]. The presence of 5′ cap and 5′ UTR will provide stability and functionality to the proposed mRNA, and the presence of the Kozak sequence will enable the vaccine to start the translation process in eukaryotic cells [44]. Immunogenicity, effectiveness, efficacy, and half-life of the vaccine are aided by adjuvants [45]. Human beta-defensin (HBD1) was added to co-stimulate the vaccine’s capacity to elicit an antigenic response [23]. MITD helps to connect the CTL epitopes to the MHC-I segment in the endoplasmic reticulum so that it can be recognized by antigen-presenting cells. The addition of a plasminogen activator, tPA, helps translate mRNA out of the host immune cells. Additionally, aiding in stability and translation initiation are the 3′ UTR and the polyA tail appended to the 3′ ends of the designed mRNA [46].

The physicochemical properties of the vaccine design show that it is a viable candidate for the Mokola virus vaccination. The mRNA vaccine design has a molecular weight (MW) of 129190.83 Da (129 kDa) and an estimated theoretical pI of 8.58 which is quite similar to our previous study on “mRNA vaccine design for Epstein–Barr virus: an immunoinformatic approach” which has approximately 118 kDa and 8.31 theoretical pI [47]. Also, these values suggest that the vaccine is basic and falls within the same range as a comparable vaccine designed by Ahammad and Lira in the study titled “Designing a novel mRNA vaccine against SARS-CoV-2: An immunoinformatics approach” [48]. The protein is stable based on the instability index score of 39.43. The construct is thermostable, as indicated by its aliphatic index of 74.81 [49]. The GRAVY (Grand Average of Hydropathicity) score measures a protein’s hydrophobicity or hydrophilicity. The mRNA GRAVY score of -0.478 suggests that the protein is relatively hydrophilic. This generally indicates that the protein is likely to be soluble in aqueous environments, which is advantageous for vaccine formulation as soluble proteins are easier to handle and less likely to form aggregates [50].

Our vaccine’s secondary structure analysis revealed that its globular shape, excellent flexibility, and stability are all features of the developed vaccine. The Ramachandran plot indicates that the vaccine-construct amino acid is located in the most favored region, with a coverage of 76%, indicating that the quality of the predicted model is good. This result aligns with the study of Aiman et al.. (2024) on “Immunoinformatic-guided novel mRNA vaccine designing to elicit immunogenic responses against the endemic Monkeypox virus” which indicates the coverage of a corresponding amino acids in the most favorable region [51]. The low energy score from the docking of the ligand and receptor suggests that the vaccine design exhibits a high binding affinity for TLR-3. Strong affinities between the molecules are indicated by this low binding energy score [52]. The molecular dynamics simulation technique was used to check the atomic stability of the vaccine with TLR and our corresponding result revealed that it was favorable. The in silico immunological simulation’s demonstration of the development of an immune response suggests that the antigen was administered along with the relevant antibodies. Additional in vivo and in vitro testing is required to confirm the proposed mRNA vaccine’s potential against the Mokola virus’s antigenic proteins. This will help to validate the vaccine’s effectiveness and potency in eliciting an immune response against the virus.

## Conclusions

This study has designed an mRNA vaccine against the five antigenic proteins of Mokola virus using in-silico techniques. The vaccine is ascertained to be antigenic, non-toxic, and non-allergic, which may be further subjected to in-vitro and in-vivo studies.

## Data Availability

All dataset analyzed in this research are all publicly available at the National Centre of Biotechnology Information (NCBI) and could be made available upon request.

## Abbreviations

MOKV: Mokola Virus
NCBI: National Center for Biotechnology Information
CTL: CD8 + T lymphocytes
HTL: CD4 + T lymphocytes
TLR: Toll-like Receptor
MHC-I: Major Histocompatibility Complex
MHC-II: Major Histocompatibility Complex
HBD1: Human Beta Defensin 1
GRAVY: Grand Average of Hydropathicity
BLAST: Basic Local Alignment Search Tool
NK Cells: Natural Killer Cells
mRNA: Messenger Ribonucleic Acid
